# Supporting the Development of Evidence-Informed Policy Options: An Economic Evaluation of Hypertension Management in Ghana

**DOI:** 10.1016/j.jval.2019.09.2749

**Published:** 2020-02

**Authors:** Mohamed Gad, Johanne Lord, Kalipso Chalkidou, Brian Asare, Martha Gyansa Lutterodt, Francis Ruiz

**Affiliations:** 1 Global Health Development group, School of Public Health, Imperial College London, International Decision Support Initiative, London, England, UK; 2 Southampton Health Technology Assessments Centre, University of Southampton, Southampton, England, UK; 3 Ghana National Drugs Programme, Ministry of Health, Accra, Ghana; 4 Pharmaceutical Services, Ministry of Health, Accra, Ghana

**Keywords:** cardiovascular, cost-effectiveness analysis, evidence-based decision making, Ghana, health technology assessment, HTA, hypertension, UHC, universal health coverage

## Abstract

**Objectives:**

Universal healthcare coverage in low- and middle-income countries requires challenging resource allocation decisions. Health technology assessment is one important tool to support such decision making. The International Decision Support Initiative worked with the Ghanaian Ministry of Health to strengthen health technology assessment capacity building, identifying hypertension as a priority topic area for a relevant case study.

**Methods:**

Based on guidance from a national technical working group of researchers and policy makers, an economic evaluation and budget impact analysis were undertaken for the main antihypertensive medicines used for uncomplicated, essential hypertension. The analysis aimed to address specific policy questions relevant to the National Health Insurance Scheme.

**Results:**

The evaluation found that first-line management of essential hypertension with diuretics has an incremental cost per disability-adjusted life-year avoided of GH¢ 276 ($179 in 2017, 4% of gross national income per capita) compared with no treatment. Calcium channel blockers were more effective than diuretics but at a higher incremental cost: GH¢ 11 061 per disability-adjusted life-year avoided ($7189 in 2017; 160% of gross national income per capita). Diuretics provide better health outcomes at a lower cost than angiotensin-converting enzyme inhibitors, angiotensin receptor blockers, or beta-blockers. Budget impact analysis highlighted the potential for cost saving through enhanced price negotiation and increased use of better-value drugs. We also illustrate how savings could be reinvested to improve population health.

**Conclusions:**

Economic evaluation enabled decision makers to assess hypertension medicines in a Ghanaian context and estimate the impact of using such evidence to change policy. This study contributes to addressing challenges associated with the drive for universal healthcare coverage in the context of constrained budgets.

## Highlights

• This article provides details of a technical modeling work collaboratively built in Ghana with a high-level policy orientation.
• Recent developments in Ghana further highlight the constraints imposed on current resources available for health.
• This is the first national health technology assessment study to be commissioned and conducted under Ghanaian government supervision. There are no other studies that offer the same level of contextualized information to support the National Health Insurance Authority and the Ghanaian Ministry of Health in their policy-making efforts toward improving value for money in hypertension management.
• A range of cost-saving and health-improving policy scenarios were identified that could have a major impact on reducing the burden of hypertension and contribute to the financial sustainability of the National Health Insurance Scheme.

## Introduction

Ghana is a West African lower-middle-income country with an estimated gross national income per capita of $4490 in 2017.^1^ The country has a long-standing commitment to achieving universal healthcare coverage (UHC). In 2003, Ghana was the first Sub-Saharan African country to introduce the National Health Insurance Scheme (NHIS),^2^ aiming to improve access to services and promote better health outcomes for the 28.2 million Ghanaians.^3^ The NHIS, which represents a significant milestone on the country’s path toward UHC, claims to cover more than 95% of disease conditions currently prevalent in Ghana,^2^ with some studies suggesting that the NHIS has reduced out-of-pocket expenditure.^4^

Despite this progress, the NHIS faces considerable challenges relating to its financial sustainability. For example, more than 60% of NHIS members are exempt from paying full premiums. In addition, provider payment delays lead major providers to threaten to leave the scheme, causing a regular interruption of drug supplies.^5^

As Ghana transitions away from development assistance funds, its co-financing obligations are expected to rise.^6^^,^^7^ Consequently, the challenge of NHIS financing is expected to escalate and grow more complex as budgetary constraints are forced to be confronted in the absence of donor support in major disease areas. This is likely to be more difficult given the potential loss of relevant technical capacity as donors depart. The future of the NHIS may well depend on restructuring its financing mechanisms and adjusting its policies and coverage decisions. In this context, the use of resource allocation tools, such as health technology assessment (HTA), combined with carefully designed quality improvement strategies, may be critical.

Despite a growing economy, the increase in national income may not necessarily translate into better health outcomes. The changes needed to ensure the NHIS is financially sustainable and progress on UHC is maintained raise urgent technical, institutional, and political challenges. Sustaining the current system and progressing toward UHC in Ghana may well depend on the system’s ability to make some tough trade-offs. This can be achieved only with an evidence-formed, transparent, deliberative (and therefore defensible) decision-making process.

## Aim and Objectives

This article describes an initial attempt to support the use of evidence in achieving better value for money as part of an ongoing process to institutionalize HTA. A multistakeholder technical working group (TWG) was convened in 2016, with members from across the government, health insurance, providers, academics, and civil society. This TWG provided strategic leadership in the development of a policy oriented toward economic evaluation focusing on a high-priority disease area (hypertension) as a case study on HTA. International support was provided by the Global Health and Development (GHD) group (formerly NICE International) at Imperial College London and the University of Southampton HTA center.

Hypertension was highlighted as a top priority by the Ghanaian Ministry of Health and National Health Insurance Authority (NHIA). Cerebrovascular events, ischemic heart disease, and diabetes mellitus occupy the second-, third-, and seventh-ranked causes of death in Ghana, respectively.^8^ Hypertension is a prominent risk factor for these conditions and for other noncommunicable diseases. It is widely recognized that better control of high blood pressure can save lives and money. Notably, hypertension is ranked first among the 5 leading global risks for mortality.^9^

Focusing on the assessment of the cost-effectiveness of 5 main classes of medicines for the treatment of uncomplicated essential hypertension, we modeled a number of policy scenarios and provide a budget impact analysis from the perspective of the NHIS. We believe that the issues raised in this article will resonate with policy makers from low- and middle-income countries in a similar situation as Ghana.

The model results were used to update the 2017 Standard Treatment Guidelines, which drive prescribing and reimbursement across Ghana.^10^ Antihypertension medicines are now sequenced for prescribing based on our analysis, a prospect that was not available in previous versions of the guidance. Furthermore, the model findings enabled the Ministry of Health to start a drug price negotiation process resulting in more efficient procurement. As a result of this experience, a drug-pricing committee has been established in Ghana as one major client of such analyses (M. Gyansa-Lutterodt, Ministry of Health, oral communication, March 2019). For further insights on the impact in Ghana, please refer to other International Decision Support Initiative resources including the website and the Ghana learning review.^11^^,^^12^

## Methods

### Population and Subgroups

The analysis focused on adults aged 20 years and older with uncomplicated primary (essential) hypertension as a target population. We did not consider hypertension secondary to clinical conditions (such as kidney disease or endocrine disorder) or hypertension during pregnancy.

Hypertension was defined as systolic blood pressure of 140 mm Hg or greater or diastolic blood pressure of 90 mm Hg or greater, with 4 levels of severity: controlled (<140/90 on treatment), mild (140-159/90-99), moderate (160-179/100-109), and severe (180+/110+).

The population was stratified by sex, age (6, 10-year bands), level of blood pressure (normal, mild, moderate, and severe), hypertension awareness, and treatment status (treated, aware but not treated, and not aware). This defined 120 subgroups with hypertension for the population in question.

### Study Perspective

A third-party payer/health system (NHIS) perspective was adopted in line with the policy context in undertaking this work. The Ghanaian 2010 Standard Treatment Guidelines (STGs) note appropriate indications and contraindications for the drug classes but do not make specific recommendations about which class or combination should be preferred for particular groups of patients. Our aim was to estimate future healthcare costs and clinical and patient outcomes associated with the STG treatment options to inform more specific recommendations about clinically significant, cost-effective, and affordable drug choices for NHIS coverage decisions.

### Comparators and Policy Options

A core treatment model was used to estimate the long-term costs and health effects of the 5 main classes of antihypertensive drugs and a “no intervention” comparator:

1. angiotensin-converting enzyme (ACE) inhibitors
2. angiotensin receptor blockers (ARBs)
3. beta-blockers (BBs)
4. calcium channel blockers (CCBs)
5. thiazide-like diuretics (TZDs)
6. no intervention (NI)

We excluded other agents because they are not commonly used for patients with primary hypertension.^13^ For simplicity, we did not model sequential or multiple treatment combinations.

We also modeled a broader range of policy options, including the following:

• mechanisms to increase the use of more cost-effective antihypertensive drugs (eg, changes to essential drug listing, negotiation of price reductions, or more prescriptive STG recommendations)
• active case finding to identify people with high blood pressure to refer for diagnosis and appropriate treatment (eg, opportunistic screening in pharmacies^14^)

The particular scenarios evaluated in the pilot study are described in the “Results” section.

### Model Structure and Assumptions

**Figure 1:**
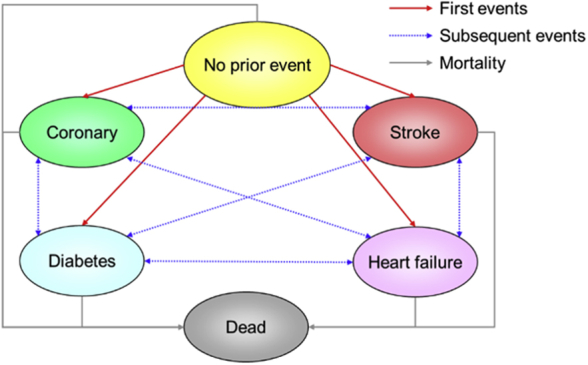
Structure of the hypertension core treatment model.

The structure of the core treatment model is illustrated in Figure 1. Patients start in the “no prior event” state and over time may experience 1 or more adverse event. At any point in time, patients must be in 1 and only 1 of the 6 health states, but they can move between health states in successive time periods as new events occur. If patients survive a first event, they are then at increased risk of a second event.

### Time Horizon and Cycle Length

The model uses a cycle length of 1 year. Patients’ risks of an event depend on their current health state and also risk factors including age, sex, and severity of hypertension. Two costs are associated with each nonfatal event: 1 for treatment in the first cycle after the event and 1 for subsequent care for each cycle in which the patient remains in the health state. Costs are therefore high in the first year after a stroke, as they include costs for acute admissions and rehabilitation. If the patient survives for the first year, ongoing costs for outpatient follow-up and preventive treatment are lower. The time horizon chosen for this analysis was that of a lifetime based on an upper limit of achievable life expectancy. Both costs and outcomes were discounted at 3% per year. Because of the absence of locally adopted discount rates, 3% was chosen based on international literature recommendations, keeping the rate constant over time.^15^ All results are displayed in 2017 Ghana Cedis (GH¢; $1.5387, purchasing power parity exchange rate).^1^

### Health Outcomes

The model estimates the number of clinical events expected for a defined population over a lifetime time horizon under each comparator. The included events were chosen to reflect the main negative consequences of high blood pressure (nonfatal acute coronary events and strokes, and mortality) and also events for which some antihypertensive drugs have a protective effect (new onset of diabetes and heart failure). The impact of these clinical events on individuals was quantified using disability-adjusted life-years (DALYs).^16^^,^^17^

### Model Inputs

#### Population and baseline risks

The total size of the Ghanaian population by sex and age was estimated from the 2010 census.^18^ The prevalence of hypertension in Ghana was taken from the 2014 Ghana Demographic and Health Survey.^19^^,^^20^ The prevalence of hypertension in children and teenagers was assumed to be negligible; hence, we excluded individuals younger than 20 years.

The annual probabilities of the first incidence of coronary heart disease (CHD), stroke, heart failure, and diabetes for each subgroup in the absence of treatment were estimated from international data.^21^, ^22^, ^23^, ^24^, ^25^ The baseline risks of CHD, stroke, heart failure, diabetes, and all-cause mortality were assumed to double after an initial nonfatal event.

#### Treatment effects and transition probabilities

The effects of antihypertensive treatment were estimated from high-quality meta-analyses of international trial data. There is a good evidence that the effects of the main classes of antihypertensive drugs vary by ethnicity and by relative effects on clinical endpoints.^21^^,^^26^^,^^27^ The model therefore estimates the effectiveness of a medication using a 3-step process that combines the estimates of the effect of antihypertensive class on blood pressure lowering in black patients,^28^ then estimates the overall effect of blood pressure lowering on the incidence of adverse outcomes, and then estimates the relative effect of a different antihypertensive class on these outcomes.

#### Resource use and costs

##### Medication costs

The costs of antihypertensive medications are based on the NHIS price for drugs on the essential medicines list (extracted in 2017 and assuming a daily dose as recommended in the Ghanaian STGs (median of range).^13^

Based on the NHIS price and estimated use within class, the mean cost per year ranged from GH¢ 26 per year for diuretics to GH¢ 399 per year for CCBs. In sensitivity analysis, we also tested the impact of using the least and most expensive drug and formulation within each class.

##### Adverse event costs

The unit costs of services were based on a weighted average of NHIS tariffs for public hospitals, private hospitals, and tertiary hospitals.^29^, ^30^, ^31^ For the base-case analysis, we assumed a distribution of 40%, 40%, and 20% for public, private, and tertiary hospitals, respectively. The analysis assumes a ceiling of current NHIS coverage at 42% with an average utilization rate of 80% for those insured.^32^ Other sources of data on resource use and clinical management include NHIA diagnosis-related group schedule data and, where necessary, clinical judgment.

The package of services for stroke was based on recommended outpatient follow-up every 2 weeks for 4 times after discharge, then every month for 3 times, then every 6 months for at least 3 years. We assumed a similar follow-up after acute admission for CHD and heart failure.

##### Valuation of health outcomes

Health outcomes were summarized in the form of DALYs. Years of life lost by age are based on standard life expectancy as set by the World Health Organization’s (WHO’s) 2017 global health estimates,^33^ discounted at 3% per year. Disability weights for CHD, stroke, heart failure, and type 2 diabetes were 0.124, 0.266, 0.201, and 0.015, respectively, from the 2003 WHO estimates.^17^ More recent estimates are not available at the level required for the model, for example, as an average for all people surviving a stroke.^34^ In the base case, the model uses the default constant of 0.1658. Refer Table 1 for detailed data sources of selected parameters and their respective references.

**Table 1:** Sources of model inputs.

Parameter type	Data source	Reference
Target population, prevalence, and baseline risks		
Population	• Ghana 2010 Population & Housing Census: Summary of Report of Final Results, 2012	[25]
Prevalence of hypertension	• Ghana Demographic and Health Survey (GDHS 2014)	[26]
Baseline risks of adverse events • Annual probabilities of first incidence of CHD, stroke, heart failure, and diabetes for each subgroup in the absence of treatment	• Multivariate analysis of primary care data for black African patients living in the United Kingdom for predicting cardiovascular risk (QRISK2) and incidence of type 2 diabetes (QDscore) • Relative incidence of CHD, stroke, and heart failure by age (Singh et al^22^) and by severity of hypertension (Ettehad et al^21^) • All-cause mortality (WHO Ghana life table 2015)	[27, 28, 29, 30, 31]
Treatment effects		
Effects of main classes of drugs by ethnicity, blood pressure lowering, and incidence of diabetes	• Meta-analyses of international trial data by ethnic group • Estimates of the mean reduction in systolic blood pressure by drug class in black patients • Summary estimates of the effects of blood pressure lowering on the incidence of different endpoints: CHD, stroke, heart failure, and all-cause mortality • Effects on the incidence of new onset of diabetes	[27,33,34]
DALY loss per event		
Years of life lost by age of death Disability weights	• WHO estimates for disability weights, from Global Burden of Disease 2004 • WHO standard life expectancy 2015	[41,42]
Resource use and costs input		
Medication cost (currency: Ghana cedis [GHC], 2016)	• NHIS price for drugs on the essential medicines list, dosage as recommended by Ghanaian STGs	[43,44]
Adverse event cost	• Based on weighted average of NHIS tariffs for public, private, and tertiary hospitals	[35, 36, 37]

CHD indicates coronary heart Disease; DALY, disability-adjusted life-year; GDHS, Ghana Demographic and Health Survey; GH¢, Ghana cedis; NHIS, National Health Insurance Scheme; STGs, Standard Treatment Guidelines; WHO, World Health Organization.

### Analyses

Analyses were carried out in Microsoft Excel 2013. The model is available under a Creative Commons Attribution-Non Commercial-Share Alike License (CC BY-NC-SA 4.0). Long-term estimates of cost-effectiveness (incremental cost per DALY avoided) and budget impact (over a 5-year period) were presented. Model estimates were available for the whole hypertensive population or for particular cohorts (eg, only patients currently receiving treatment).

Uncertainty was explored both deterministically and probabilistically.^35^ Further details on model inputs (which include assigned distributions on relevant parameters) can be found in the supplementary material (found at https://doi.org/10.1016/j.jval.2019.09.2749).

## Results

### Base-Case Results

The model estimated that there are 2.8 million Ghanaians with hypertension, of whom about one-fifth (0.5 million) have their blood pressure effectively controlled with medication. Of those with severe hypertension who are at the greatest risk of an adverse event such as a stroke or a myocardial infarction, 82% are unaware of their condition, and a further 4% are not receiving any treatment.

Results are presented for the approximately 340 000 people estimated to be covered by the NHIS (based on 42% NHI coverage rates) and receiving treatment for hypertension, including those with adequately controlled blood pressure and those with mild, moderate, and severely raised blood pressure despite treatment. About 35% of those on treatment do not have good blood pressure control, and more than 17 000 have severe disease despite treatment.

**Figure 2:**
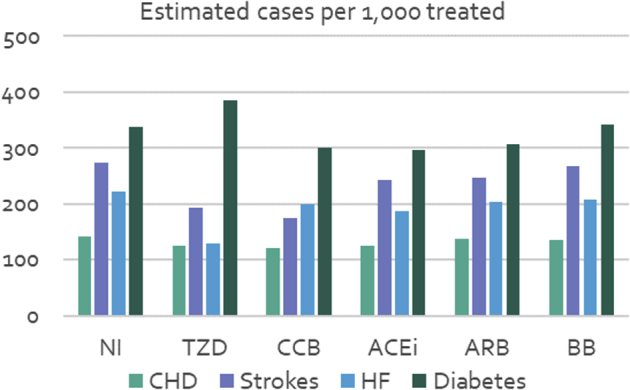
Estimated number of adverse events in the treated population (lifetime incidence).

The estimated numbers of adverse events over the time horizon per 1000 patients treated are shown in Figure 2. Compared with NI, all classes of antihypertensive medications are expected to reduce the number of coronary events, strokes, and incident cases of heart failure. CCBs, ACE inhibitors, and ARBs are also expected to reduce the incidence of type 2 diabetes, but TZDs and BBs are estimated to increase the diabetes incidence. CCBs are most effective at preventing coronary events and strokes, although TZDs are better at preventing heart failure**.**

ACEi indicates angiotension-converting enzyme inhibitor; ARB, angiotension receptor blocker; BB, beta-blocker; CCB calcium channel blocker; CHD, coronary heart disease; HF, heart failure; NI, no intervention; TZD, thiazide-like diuretic.

The additional costs and DALYs avoided for each drug class compared with NI are shown in Table 2. TZDs resulted in an additional cost of about GH¢ 300 000 per 1000 patients treated and about 1000 additional DALYs avoided compared with NI, giving an incremental cost-effectiveness ratio (ICER) of GH¢ 276 per DALY avoided. Using a CCB rather than diuretic costs an additional GH¢ 5.2 million and avoids a further 471 DALYs, giving an ICER of more than GH¢ 11 000 per DALY avoided. ACE inhibitors, ARBs, and BBs were estimated to be more costly and less effective (fewer DALYs avoided) than TZDs.

**Table 2:** Incremental cost-effectiveness analysis: per 1000 treated population.

	Total		Incremental (compared with no intervention)		ICER (compared with next best alternative)	
	Cost (GH¢)	DALYs	Cost (GH¢)	DALYs avoided	ICER (GH¢ per DALY avoided)	
NI	536 562	13 447	—	—	—	
TZD	827 495	12 394	290 933	1052	276	vs NI
CCB	6 034 688	1523	5 498 126	1523	11 061	vs TZD
ACEi	5 383 737	690	4 847 175	690	Dominated	
ARB	3 934 709	416	3 398 147	416	Dominated	
BB	1 871 136	202	1 334 573	202	Dominated	

ACEi indicates angiotension converting enzyme inhibitor; ARB, angiotension receptor blocker; BB, beta-blockers; CCB, calcium channel blockers; DALYs, disability-adjusted life-years; GH¢, Ghana cedis; ICER, incremental cost-effectiveness ratio; NI, no intervention; TZD, thiazide-like diuretics.

The estimated impact on the NHIS budget is shown in Table 3. For the whole NHIS-covered population treated for hypertension, the estimated cost of TZD would be GH¢ 28.9 million over 5 years compared with NI. The additional cost of prescribing a CCB is much higher (more than GH¢ 531 million over 5 years).

**Table 3:** NHIS budget impact for the entire treated population (343 488 patients).

Total costs (GH¢ undiscounted)						
	Year 1	Year 2	Year 3	Year 4	Year 5	Total
NI	5 507 598	6 453 082	7 102 141	7 549 956	7 861 999	
TZD	8 426 749	13 312 890	13 688 062	13 925 951	14 067 494	
CCB	71 468 372	135 651 998	132 349 908	129 102 548	125 960 627	
ACEI	66 093 318	124 245 912	120 836 360	117 496 649	114 292 309	
ARB	48 538 500	89 934 993	87 600 771	85 267 258	83 003 255	
BB	22 496 680	39 411 909	38 867 829	38 203 087	37 485 339	
TZD vs NI	2 919 150	6 859 808	6 585 921	6 375 995	6 205 495	28 946 370
CCB vs TZD	63 041 623	122 339 108	118 661 846	115 176 597	111 893 132	531 112 307

ACEi indicates angiotension-converting enzyme inhibitor; ARB, angiotension receptor blocker; BB, beta-blocker; CCB, calcium channel blocker; GH¢, Ghana cedis; NI, no intervention; TZD, thiazide-like diuretic.

### Probabilistic Sensitivity Analysis

We used probabilistic sensitivity analysis to assess the impact of uncertainties over input parameters, including the prevalence of hypertension by treatment status, baseline risks of cardiovascular events (CHD and stroke) and onset of heart failure and type 2 diabetes, effectiveness of treatment with the 5 classes of antihypertensives, and level of use of NHIS services. Details are available in the supplementary material.

Based on 1000 probabilistic sensitivity analysis iterations, the ICER for diuretics compared with NI was estimated at GH¢ 289 per DALY avoided (95% of iterations provided estimates between GH¢ 244 and GH¢ 335 per DALY avoided), and the ICER for CCBs compared with diuretics was 10 964 (95% from GH¢ 9043 to GH¢ 13 314 per DALY avoided).

The cost-effectiveness acceptability curve for the most cost-effective options is available in the supplementary material. Below a willingness-to-pay threshold of GH¢ 200 per DALY avoided, the probability that any antihypertensive treatment is cost-effective is negligible. Between a threshold of about GH¢ 400 and 8600 per DALY avoided, it appears almost certain that diuretics are the most cost-effective option. At greater than GH¢ 8600 per DALY avoided (about twice the gross national income per capita), the probability that CCBs are cost-effective begins to rise, reaching 100% of simulations at GH¢ 15 100 per DALY avoided.

### Policy Scenarios

We also explored 5 policy scenarios identified by the TWG. The options considered (see Table 4) include cost-saving possibilities (eg, lower prices or shifting from more expensive pharmaceutical options to less expensive ones when clinically appropriate). For illustration, we also show how resulting savings could be reinvested in health-improving scenarios to increase coverage or reduce the number of undiagnosed, untreated, or inadequately treated patients. To model scenario 5, estimates from a trial of community pharmacy-based screening in Ghana were used.^14^

**Table 4:** Results of cost-saving and health-improving scenarios.

Scenario	Patients changing drugs	DALYs avoided (discounted)	Lifetime cost saving to NHIS, GH¢ millions (discounted)	Cost savings (vs current practice), GH¢ millions (undiscounted)					
				Year 1	Year 2	Year 3	Year 4	Year 5	Total 1-5
1. 10% cut in mean drug prices	0	0	93.7	3.3	6.5	6.3	6.1	5.9	28.0
2. 10% shift from ACEi/ARB/BB to TZD	6050	3471	19.1	0.7	1.4	1.3	1.3	1.2	5.9
3. 10% shift from CCB to TZD	13 033	–6135	67.9	2.4	4.6	4.5	4.4	4.2	20.2
4. Prescribe TZD to 10% of patients diagnosed with hypertension who are not currently treated	9170	10 776	2.16	0.06	0.16	0.15	0.14	0.14	0.51
5. Offer screening to 5% of NHIS patients older than 40 y without a diagnosis of hypertension	104 476 invited for screening, 71 044 screened, 8997 offered TZD	5512	−5.07	−4.20	−0.07	−0.07	−0.06	−0.06	−4.47

ACEi indicates angiotension-converting enzyme inhibitor; ARB, angiotension receptor blocker; BB, beta-blocker; CCB, calcium channel blocker; DALYs, disability-adjusted life-years*;* GH¢, Ghana cedis; NHIA, National Health Insurance Authority; NHIS, National Health Insurance Scheme; TZD, thiazide-like diuretic.

### Cost-Saving Scenarios

The cost-saving scenarios in Table 4 were modeled on the cohort of 343 488 patients covered by the NHIS (based on 42% coverage) who are estimated to be currently receiving antihypertensive medication. The results show substantial potential for cost savings for all 3 scenarios considered (see Table 4). Scenario 1 (10% reduction in mean drug cost) would yield the greatest savings, more than GH¢ 28 million over the first 5 years. This was followed by scenario 3 (10% shift from CCB to TZD) with 5-year savings of more than GH¢ 20 million, although this would be accompanied by a deterioration in health outcomes. In contrast, scenario 2 (10% shift from ACE inhibitors/ARBs/BBs to TZDs) yields a 5-year savings of about GH¢ 6 million in addition to increased health benefits.

### Health-Improving Scenarios

The health-improving scenarios in Table 4 were modeled each on their respective cohort of patients. Results (see Table 4 ) indicated that prescribing diuretics to 10% of currently untreated NHIS members with a diagnosis of hypertension (scenario 4) would cost an additional GH¢ 0.5 million over 5 years but yield a gain of more than 10 700 DALYs avoided. A more ambitious program to screen 5% of NHIS members older than 40 years (scenario 5) would cost about GH¢ 4.47 million over 5 years for a gain of about 5500 DALYs avoided.

## Discussion

The analysis shows that in the Ghanaian context, diuretics are dominant when compared with ACE inhibitor, ARB, and BB drug classes for first-line treatment of uncomplicated hypertension (see Table 2; ie, diuretics provide better health outcomes at a lower cost from an NHIS perspective). This result is driven by a greater reduction in stroke incidence. CCBs were estimated to give greater protection against stroke and new-onset diabetes than diuretics, although they are more expensive and associated with a greater incidence of heart failure (see Fig. 2). Compared with no treatment, diuretics cost an additional GH¢ 276 per DALY avoided. The incremental cost per DALY avoided for CCBs compared with diuretics was much higher at GH¢ 11 061. Although differences in the estimated number of cases of CHD and stroke per 1000 treated with diuretics and CCBs are marginal (both reduce incidence), there is a much larger difference between the 2 classes in terms of onset of diabetes and heart failure. According to the model, thiazide use is associated with 85 more cases of diabetes per 1000 patients treated than CCBs, whereas CCB use is associated with 69 more cases of heart failure per 1000 persons treated than diuretics.

The results of this study are consistent with those of the findings of National Institute for Health and Care Excellence guideline on hypertension in primary care, in which CCBs and diuretics dominated the other antihypertension classes (BBs, ACEs, and ARBs). Note, however, that the UK study focused on a subgroup of 65-year-old men and women with an annual cardiovascular disease risk of 2%, heart failure risk of 1%, and diabetes risk of 1.1%.^36^

It is important to highlight that in the absence of a legitimate and robustly estimated country-specific willingness-to-pay threshold value, the true opportunity costs of selecting interventions from various disease areas remain unexplored.^37^ Nevertheless, we note that our results suggest that hypertension treatment with diuretics and CCBs are cost-effective compared with other treatments proposed for other disease areas. In the Zelle et al^38^ study, for example, which looked at the cost-effectiveness of various breast cancer control options, it would seem that TZDs and CCBs are at least as cost-effective as screening by clinical breast examination or undertaking an awareness-raising campaign using mass media and substantially more cost-effective than mammography screening of women aged 40 to 69 years (see Table 8 in Section B - Discussion in Supplementary Material 1 found at https://doi.org/10.1016/j.jval.2019.09.2749 for further details).

### Pricing and Procurement

The cost-effectiveness results were highly sensitive to assumptions about the price of drug formulations. For example, if the lowest-priced CCB is used (generic amlodipine 10-mg tablet, GH¢ 52 per year), rather than the NHIS median (GH¢ 399 per year), the ICER for CCB versus diuretic falls to GH¢ 806 per DALY avoided. Conversely, the most expensive CCB covered by the NHIS (branded amlodipine 5 mg, at GH¢ 2738 per year) has an ICER of more than GH¢ 80 231 per DALY avoided. Similar estimates have been calculated for thiazides (see Table 9 in Section B - Discussion in Supplementary Material 1 found at https://doi.org/10.1016/j.jval.2019.09.2749). This highlights the importance of implementing more effective mechanisms aimed at pricing and procurement. The analysis suggests there are significant potential savings by switching to lower-priced formulations or negotiating lower prices for medicines. This generates potential savings in budget impact represented by an 83% drop in budget impact value for CCBs and 35% for thiazides.

A further impetus to improve pricing and procurement policy is provided by the observation that higher-income countries may be securing better value for money for the same medications. For example, it would be appear that nifedipine 10-mg capsules, which are priced at GH¢ 0.49/capsule in Ghana, are more expensive than the lowest-priced equivalent option available in the United Kingdom (GH¢ 0.34/capsule, based on the National Health Service indicative price for a generic drug; £1= GH¢ 6.15).^39^^,^^40^ This signals the need for more systematic, value-based price negotiations to bring down costs and increase the availability of essential medicines.^41^

### NHIS Prescription Patterns and Utilization

Medication costs, rather than the cost of care, appear to be the main drivers of total costs in this model. Although drug price negotiations can yield major savings for the NHIS, it is indeed possible to achieve significant savings by implementing appropriate prescribing rules and restrictions. The Ghanaian 2010 STGs did not specify any clinically informed preferences or rankings for lines of management of uncomplicated essential hypertension using different classes^13^ based on subgroups of the treated population. In contrast, in the United Kingdom, the recommendation is to initiate therapy with diuretics or CCBs for those aged 65 or older or black patients of any age.^42^ Moreover, current NHIS policies appear not to encourage the prescription of lower-priced formulations or better-value classes over others, instead providing comprehensive coverage policy across various classes and formulations. Nevertheless, the seventh edition of STGs released in 2017 has now incorporated a preferential approach to hypertension treatment based on this study and an available international clinical guidance.^10^

If CCBs are to be provided for the entire NHIS-covered hypertensive population, the model estimates the impact on the NHIS budget to be greater than GH¢ 480 million over 5 years, which is more than 18-fold higher than if diuretics were provided instead.

### Limitations

This study has a number of potential limitations. For instance, the model deals only with monotherapy and does not explore sequential or combination therapy. There are several reasons for this. First, it is not clear to what extent combination treatment is currently used in Ghana or whether this would greatly enhance the cost-effectiveness of national policy on antihypertensive treatment. Furthermore, from an epidemiological point of view, the main morbidity and mortality outcomes do not differ greatly between treatment groups when drugs were combined, and the European Medicines Agency advises that monotherapy is sufficient for initiating treatment up until the level of mild hypertension.^28^^,^^43^ As such, our main objective was to identify differences across individual drug classes and provide information on how best to initiate treatment.

The model in its current format includes only NHIA costs, including the costs of antihypertension medications and (where possible) the cost of policy implementation, in addition to the cost of diagnosis, treatment, and care for adverse events. Out-of-pocket, informal caring and productivity costs, although important for patients and families, are not included in this version of the model. This is highly recommended as a topic for further study in future versions of the model.

Estimates of health service utilization were based on NHIS guidance and clinical judgment, rather than empirical evidence. Furthermore, the unit costs of services were based on a weighted average of NHIS tariffs for public hospitals, private hospitals, and tertiary hospitals).^29^, ^30^, ^31^ For the base-case analysis, in absence of actual utilization data, we assumed a distribution of 40%, 40%, and 20% for public, private, and tertiary hospitals, respectively.

Disability weights for CHD, stroke, heart failure, and type 2 diabetes were extracted from 2003 WHO estimates.^16^ More recent estimates are not available at the level required for the model, for example, as an average for all people surviving a stroke.^34^ Furthermore, the annual probabilities of first incidence of CHD, stroke, heart failure, and diabetes for each subgroup were estimated from international data. It would have been preferable to use estimates of incidence from Ghana or other West African countries with a similar population and healthcare profile. Nevertheless, cohort studies with longitudinal follow-up of a population sample from these contexts have not been available.

The impact on equity-related issues was not explored in this model, although a future iteration could explore disaggregating the findings by relevant population group (eg, urban vs rural).^44^

## Conclusion

Achieving UHC is a goal shared by policy makers in many low- and middle-income countries. Ensuring that it can be delivered sustainably and fairly will require trade-offs to be identified and addressed head on. For many aid-dependent health systems, HTA as a means for supporting decisions around what gets covered and for whom appears to be of interest to policy makers,^44^ and yet its implementation and practical policy value have not often been shown. The present analysis, used as part of a Ghanaian HTA pilot study, has contributed to the building of in-country technical and governance capacity that will be needed to institutionalize HTA. By undertaking a comparative cost-effectiveness and budget impact analysis, and incorporating alternative policy scenarios, Ghanaian stakeholders were given hands-on experience in the assessment and appraisal. Policy makers were given information to estimate the impact of moving toward more effective prescribing policies based on the findings of the model. A range of cost-saving and health-improving scenarios were identified that could have a major impact on reducing the burden of hypertension and contribute to improving the financial sustainability of the NHIS. Replicating this approach to other high-burden disease areas in Ghana is likely to lead to extensive systemwide benefits and offers a practical way for implementing the 2014 World Health Assembly resolution on Health Intervention and Technology Assessment for accelerating progress toward UHC.^45^
