# Supplementary material for: Supporting the Development of Evidence-Informed Policy Options: An Economic Evaluation of Hypertension Management in Ghana
Source: Value Health. 2020 Feb;23(2):171–9. doi: 10.1016/j.jval.2019.09.2749 (PMC7065042; doi:10.1016/j.jval.2019.09.2749)
Supplement: Supplementary Material 1 [file mmc1.pdf]

Supplementary Material – Supporting the development of evidence-informed policy options: an economic evaluation of hypertension management in Ghana

Contents

Introduction 2
Section A – The Model 2
i. The development process 2
ii. Aims and objectives 2
iii. Population and subgroups 2
iv. Intervention 3
v. Policy Scenarios 3
vi. Outcomes 4
vii. Parameters and sources of data 4
viii. Cost effectiveness acceptability curve 13
Section B – Discussion 14
i. Comparing this Study's ICER value with other studies in the Ghanaian context 14
ii. Pricing and procurement 15
Section C- iDSI reference case assessment of Ghana hypertension model 17
Section D – Acknowledgements 24
References 25

Introduction

This appendix describes Version 3.0 of the Ghana Hypertension Model. The model was programmed in Excel by Joanne Lord, initially based on a model developed for the 2006 update of the NICE clinical guideline on hypertension [1], and adapted for Ghana following discussion and agreement of appropriate modelling assumptions and data inputs with the Technical Working Group (see part C) and Kalipso Chalkidou, Francis Ruiz, Martha Gyansa-Lutterodt and Mohamed Gad. The model is available under a Creative Commons Attribution-Non Commercial-Share Alike license (CC BY-NC-SA 4.0).

Section A – The Model

i. The development process

The hypertension model was based on an analysis developed initially for the 2006 update of the UK, National Institute for Health and Care Excellence (NICE) clinical guideline on hypertension [1], updated and adapted for the Ghanaian context and extended to address a wider range of questions. In developing the analysis, a number of workshops and in-country visits were undertaken, supported by teleconferences and email exchanges. In partnership with the UK team, members of the Ghana-based TWG specified the decision problem to be addressed, advised on the adaptation process, the quality and relevance of data sources, the appropriateness and acceptability of assumptions and set out policy makers' decision needs.

ii. Aims and objectives

The aim of the model is to provide a platform to estimate the cost-effectiveness and budget impact of a range of interventions to prevent cardiovascular disease (CVD) through better control of hypertension in the Ghanaian population.

iii. Population and subgroups

The model estimates outcomes for patients with essential hypertension, excluding those with pre-existing CVD, diabetes and pregnant women. 48 subgroups of prevalent cases are defined by: men and women; by 6 ten-year age groups (from 20-29 to 70+); and 4 levels of blood pressure (normal due to effective control with antihypertensive medication, mild, moderate or severe; see Table 1). In addition, patients with raised blood pressure are further divided according to current treatment status (not aware of raised blood pressure; aware but not treated; treated but not controlled). Thus, in total the model includes 120 subgroups of patients.

Blood pressure status	Systolic (mmHg)		Diastolic (mmHg)
Optimal	<120	and	<80
Normal	120-129	or	80-84
High normal	130-139	or	85-89
Level of hypertension			
Grade 1, mild	140-159	or	90-99
Grade 2, moderate	160-179	or	100-109
Grade 3, severe	180+	or	110+

Table 1 Classification of hypertension by severity

iv. Intervention

The model can be used to compare expected outcomes assuming no treatment or monotherapy with one of the 5 main classes of antihypertensive medication:

1. Angiotensin Converting Enzyme Inhibitor (ACEi)
2. Angiotensin Receptor Blockers (ARB)
3. Betablockers (BB)
4. Calcium Channel Blockers (CCB)
5. Thiazide like diuretics (TZD)

v. Policy Scenarios

The analyses presented in this report include the following types of policy change:

1. Price reductions for listed drugs: e.g. 10% reduction in mean price of ACEi, ARB, BB and CCB, no change in numbers of patients treated by drug class.
2. Reduced use of less effective/more expensive drugs: e.g. switch 10% of patients currently treated with ACEi, ARB and BB to TZD.
3. Increased use of better value drugs: e.g. switch 10% of patients treated with CCB to TZD.
4. Increased treatment of diagnosed patients: e.g. use of TZD for patients aware of hypertension but not currently on treatment.
5. Increased case detection: e.g. 5% of population aged 40 years and over offered screening in community pharmacy [2].

For simplicity, the policy options that were modelled assumed a one-off change, instituted in a single year for a proportion of the current prevalent population with hypertension. The model then estimates consequent health effects and costs over the remaining lifetime for those individuals. The impact of extending the policies to more patients in future years (whether members of the initial prevalent population or to new incident cases) could be estimated in separate modelled scenarios.

Note that the modelled examples do not generally assume 100% uptake of the suggested treatment changes, because it is not likely that such major shifts in practice could be achieved in a single year. In addition, there are individual factors not included in the model that mean that the changes would not be clinically appropriate for all patients. For example, treatment may reasonably differ for patients with co- morbidities: such as use of beta blockers for patients with cardiac arrhythmia or ACEi/ARB for those with heart failure. And some patients may be contra-indicated or intolerant of some antihypertensive medications.

It should also be noted that the estimated numbers of people in the modelled subgroups include people with secondary hypertension (attributable to a specific secondary cause) and also some people with pre-existing diabetes and/or cardiovascular disease. The model estimates of baseline risk and treatment effectiveness will not be accurate for these groups.

vi. Outcomes

The effects of antihypertensive treatment were estimated from high quality meta-analyses of international trial data. There is a good evidence that the effects of the main classes of antihypertensive drugs vary by ethnicity and by relative effects on clinical end points [3] [4] [5]. The model therefore estimates effectiveness of medication by a three-step process. First the mean reduction in Systolic Blood Pressure by drug class is estimated using results from the Brewster et al meta-analysis [6], the incidences of the major events included in the model are then estimated using relative risks per 10 mm Hg reduction in SBP, stratified by baseline blood pressure, from the Ettehad et al 2016 meta-analysis. (CHD, stroke, heart failure and all-cause mortality) and Elliott and Meyer 2007 meta-analysis (for onset of type 2 diabetes) [4, 5].

In addition, Disability Adjusted Life Years (DALYs) and healthcare costs associated with interventions and incident adverse events are estimated.

vii. Parameters and sources of data

Population

The number of people in the Ghanaian population by sex and included age groups (age 20 years and older) was estimated from the 2010 census (See Table 2) [7]. People under the age of 20 were not included in the model, as we assumed that the prevalence of essential hypertension in children and teenagers would be negligible.

Age group	MEN	WOMEN	Total
**20-29**	2,043,940	2,329,662	4,373,602
**30-39**	1,467,069	1,633,143	3,100,212
**40-49**	1,025,595	1,098,853	2,124,448
**50-59**	653,182	703,611	1,356,793
**60-69**	363,294	406,426	769,720
**70+**	361,709	511,952	873,661
**Total**	5,914,789	6,683,647	12,598,436

Table 2 Number of people by sex and age group (2010 Census)

The population in Ghana has grown since 2010, but for simplicity in this version of the model, the numbers have not been inflated to reflect this. we also present result for the whole nation, but it would be possible to estimate costs and effects by subgroup.

Prevalence of hypertension

The 2014 Ghana DHS included measurement of blood pressure for a representative sample of the population: including 9,396 women between the ages of 15 and 49 and 4,388 men aged 15 to 59 [8]. These individuals were also asked if they were aware of having high blood pressure and if they were receiving treatment for high blood pressure.

The proportions of men and women in each age group with controlled, mild, moderate, and severe hypertension are shown in Figure 1. A greater proportion of women with hypertension were aware of this condition than were men with hypertension. As might be expected, a greater proportion of older people had hypertension than did young people (with a steeper percentage increase per year in women).

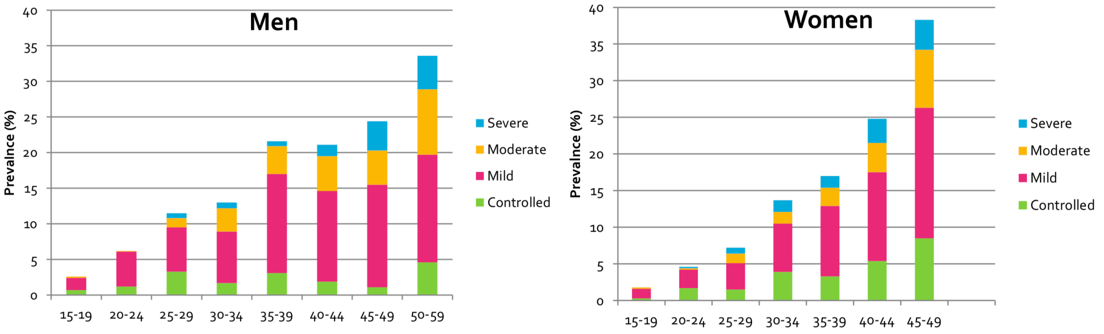

Figure 1 Prevalence of Hypertension (GDHS 2014)

The numbers of people in the 120-modelled subgroups were estimated from the data in Table 3. The prevalence of hypertension in men over the age of 60 and women over the age of 50 were estimated by simple linear extrapolation from the observed prevalence in younger age groups: 0.79% (standard error (SE): 0.05%) increase per year for men and 1.14% per year for women (SE 0.15%). The numbers of people in each subgroup were sampled probabilistically for the PSA: using beta distributions to sample the prevalence of hypertension and Dirichlet distributions to sample the split of those with controlled, mild, moderate and severe hypertension for each age/sex group.

	Treated	Not aware (NA)			Aware not treated (ANT)			Treated not controlled (TNC)		
	Controlled	Mild	Mod.	Severe	Mild	Mod.	Severe	Mild	Mod.	Severe
**MEN**										
20-29	45,989	103,785	13,090	6,545	3,620	457	228	6,034	761	381
30-39	34,564	138,737	48,176	10,148	4,840	1,681	354	8,066	2,801	590
40-49	15,902	126,221	45,591	25,300	4,403	1,590	883	7,338	2,651	1,471
50-59	30,136	90,505	55,142	28,170	3,157	1,924	983	5,262	3,206	1,638
60-69	20,052	60,220	36,690	18,744	2,101	1,280	654	3,501	2,133	1,090
70+	24,872	74,698	45,511	23,250	2,606	1,588	811	4,343	2,646	1,352
**WOMEN**										
20-29	37,275	53,933	13,262	8,841	3,424	842	561	13,697	3,368	2,245
30-39	59,120	100,194	25,333	19,897	6,361	1,608	1,263	25,446	6,434	5,053
40-49	74,825	122,537	48,152	30,558	7,780	3,057	1,940	31,121	12,229	7,761
50-59	63,555	104,081	40,899	25,955	6,608	2,597	1,648	26,433	10,387	6,592
60-69	46,922	76,842	30,195	19,163	4,879	1,917	1,217	19,515	7,669	4,867
70+	75,182	123,122	48,382	30,704	7,817	3,072	1,949	31,269	12,287	7,798

Table 3 Estimated number of people with hypertension by subgroup

Results are presented for the approximately 340,000 people estimated to be covered by the NHIS (based on 42% NHI coverage rates) and receiving treatment for hypertension, including those with adequately controlled blood pressure and those with mild, moderate and severely raised blood pressure despite treatment (see Table 4). About 35% of those on treatment do not have good blood pressure control, and over 17,000 have severe disease despite treatment.

Age group	Controlled	Mild	Moderate	Severe	Total
**MEN**					
**25**	19,315	2,534	320	160	22,329
**35**	14,517	3,388	1,176	248	19,329
**45**	6,679	3,082	1,113	618	11,492
**55**	12,657	2,210	1,347	688	16,902
**65**	8,422	1,470	896	458	11,246
**75**	10,446	1,824	1,111	568	13,949
**WOMEN**					
**25**	15,656	5,753	1,415	943	23,766
**35**	24,830	10,687	2,702	2,122	40,342
**45**	31,427	13,071	5,136	3,260	52,893
**55**	26,693	11,102	4,363	2,769	44,926
**65**	19,707	8,196	3,221	2,044	33,169
**75**	31,576	13,133	5,161	3,275	53,145
**Total**	221,925	76,451	27,960	17,151	343,488

Table 4 Estimated number of people receiving NHIS treatment for hypertension

Baseline risks of adverse events

Annual mortality rates for individuals without CVD, diabetes or heart failure were taken from the World Health Organization life table for Ghana, published in 2016 (See Table 5) [9].

	Probability per year	
Age Group	Male	Female
**25-34**	0.00449	0.00350
**35-44**	0.00599	0.00549
**45-54**	0.00996	0.00798
**55-64**	0.02032	0.01588
**65-74**	0.04975	0.04402
**75-84**	0.13624	0.12676
**85-94**	0.31228	0.29181
**95-100**	0.54465	0.51155

Table 5 All-cause mortality with no prior event (WHO Ghana life table 2016)

The annual probabilities of first incidence of CHD, stroke, heart failure and diabetes for each subgroup in the absence of treatment were estimated from international data. It would have been preferable to use estimates of incidence from Ghana, or other West African countries with a similar population and healthcare profile, but we have not been able to identify good quality cohort studies with longitudinal follow up of a population sample from these contexts.

Baseline estimates of the incidence of CVD (CHD and stroke) were taken from a multivariate analysis of primary care data for black African patients living in the UK (the QRisk2 algorithm) [10]. The derivation cohort included 6,917 individuals of Black African ethnicity, with 33 incidence CVD cases observed in 12,869-person years of follow up. The resulting estimates of incidence over ten years for men and women by age group in the absence of other risk factors are shown in Table 6. For the PSA, incidences by age/sex subgroup were sampled from beta distributions. Additional uncertainty was assumed around these

probabilistic estimates due to the extrapolation of UK data (by assuming an effective sample size of 200 per subgroup).

Age	MEN	WOMEN
**25**	0.4%	0.9%
**35**	1.9%	1.7%
**45**	4.9%	3.2%
**55**	9.2%	6.0%
**65**	15.4%	11.1%
**75**	24.9%	20.1%
**N**	**3,316**	**3,655**

Table 6 Ten-year Incidence of CVD in black African patients with no other risk factors (QRisk2)

The relative incidences of CHD, stroke and heart failure were estimated from a meta-analysis of trials of antihypertensive medications [4]. This reported 3,928, 2,220 and 1,844 incident cases of CHD, stroke and heart failure over a total 205,828-person years of follow up: proportions of coronary events and heart failure per case of CVD were 36.8% and 38.3% respectively. These proportions were sampled for the PSA using beta distributions.

The QRisk2 estimates of incidence cited in Table 6 are for people with normal blood pressure (SBP of 135) and no other risk factors. We adjusted the estimated risks of CHD, stroke and heart failure for people with mild, moderate and severe hypertension using relative risks per 10mm Hg increase in SBP from a pooled analysis of large cohort studies (see Table 7)[11]. These relative risks were sampled probabilistically from a log-normal distribution, with standard error based on the reported 95% confidence intervals.

		Mean	95% CI	
**CHD**	35-44	1.68	1.29	2.20
	45-54	1.56	1.29	1.89
	55-64	1.45	1.29	1.62
	65-74	1.33	1.29	1.38
	75-84	1.26	1.24	1.28
	85+	1.14	1.00	1.30
**Stroke**	35-44	2.05	1.89	2.22
	45-54	1.83	1.72	1.93
	55-64	1.63	1.57	1.69
	65-74	1.44	1.39	1.50
	75-84	1.28	1.21	1.35
	85+	1.10	1.03	1.18
**HF**	35-44	2.86	2.67	3.06
	45-54	2.49	2.37	2.61
	55-64	2.16	2.09	2.24
	65-74	1.88	1.82	1.94
	75-84	1.63	1.56	1.71
	85+	1.37	1.28	1.48

Table 7 Relative risk per 10mm Hg higher SBP (Singh et al 2013)

Incidence of type 2 diabetes in people with hypertension was estimated from the QDiabetes algorithm, based on a multivariate analysis of 17,057 black African patients living in the UK: see Table 8 [12]. These estimates were used for the hypertensive population in our model, irrespective of the level of blood pressure (controlled, mild, moderate or severe). The risks of diabetes in each age/sex subgroup were sampled probabilistically, assuming an effective

sample size of 500 per subgroup (less than the actual number of observed person years to increase uncertainty due to extrapolation from the UK context).

Age	MEN	WOMEN
25	0.9%	0.9%
35	4.6%	3.5%
45	12.2%	8.8%
55	21.1%	15.5%
65	25.1%	19.4%
75	22.3%	18.3%
**N**	**7,695**	**9,362**

Table 8 Incidence of type 2 diabetes in black African patients with hypertension (QDiabtes)[12]

Treatment effects
The effects of antihypertensive treatment were estimated from high quality meta-analyses of international trial data. There is good evidence that the effects of the main classes of antihypertensive drugs vary by ethnicity [3]. Brewster et al reported estimates of the mean reduction in systolic blood pressure in black patients, pooled from 26 RCTs: see Table 8 [6]. This shows that in this population, CCBs and TZD medications are more effective at reducing blood pressure.

	Reduction (mmHg)		
	Mean	95% CI	
**ACEi**	6.96	4.27	9.64
**ARB**	3.63	1.78	5.47
**BB**	3.53	-0.45	7.51
**CCB**	12.46	10.08	14.85
**TZD**	11.81	9.55	14.07

Table 9 Mean reduction in SBP for black patients (Brewster et al 2004) [6]

Ettehad et al (2016) reported summary estimates of the effects of blood pressure lowering on the incidence of different endpoints: CHD, stroke, heart failure and all-cause mortality: see Table 10 [4]. Effects on the incidence of new onset of diabetes was reported by Elliot and Meyer (2007) [5].

	RELATIVE RISK PER 10MM HG REDUCTION			SOURCE
	Mean	95% CI		
**CHD**	0.83	0.78	0.88	Ettehad et al 2016 [4]
**STROKE**	0.73	0.68	0.77	Ettehad et al 2016 [4]
**HF**	0.72	0.67	0.78	Ettehad et al 2016 [4]
**DEATH**	0.87	0.84	0.91	Ettehad et al 2016 [4]
**DIABETES**	0.87	0.75	1.01	Elliot et al 2007 [5]

Table 10 Relative risk of adverse events with blood pressure lowering

There is also evidence that different classes of antihypertensive vary in their relative effects on these endpoints: see Table 11.

		RR vs pooled comparators*			RR of outcome in black population (vs. control)
		Mean	95% CI		
**ACEi**	CHD	0.95	0.90	1.01	0.85
	Stroke	1.08	1.01	1.16	0.85
	HF	0.98	0.92	1.05	0.80
	Diabetes	0.87	0.75	1.01	0.83
	Death	1.01	0.97	1.05	0.92
**ARB**	CHD	1.06	0.98	1.15	0.96
	Stroke	0.92	0.85	0.99	0.88
	HF	0.96	0.89	1.04	0.89
	Diabetes	0.75	0.61	0.91	0.87
	Death	0.99	0.94	1.04	0.95
**BB**	CHD	1.03	0.96	1.10	0.95
	Stroke	1.24	1.14	1.35	0.97
	HF	1.04	0.93	1.16	0.91
	Diabetes	1.17	0.98	1.40	1.01
	Death	1.06	1.01	1.12	0.97
**CCB**	CHD	0.98	0.94	1.03	0.77
	Stroke	0.90	0.85	0.95	0.57
	HF	1.17	1.11	1.24	0.80
	Diabetes	0.97	0.82	1.15	0.81
	Death	0.97	0.94	1.00	0.81
**TZD**	CHD	1.02	0.97	1.09	0.82
	Stroke	0.97	0.90	1.05	0.66
	HF	0.81	0.75	0.88	0.51
	Diabetes	1.30	1.07	1.58	1.15
	Death	1.02	0.97	1.06	0.87

Table 11 Relative risk of adverse events by antihypertensive class

The model combines the above estimates of the effect of antihypertensive class on blood pressure lowering in black patients, the overall effect of blood pressure lowering on the incidence of adverse outcomes, and the relative effect of different antihypertensive class on these outcomes. These estimates are shown in the right-hand column of Table 11. Probabilistic estimates of these relative risks are sampled, based on the reported confidence intervals from the underlying meta-analysis estimates: assuming a normal distribution for the reduction in mean SBP (Table 11) and log-normal for the relative risks with blood pressure lowering (Table 10) and per antihypertensive class (Table 11).

Medication costs

The costs of antihypertensive medications are based on the NHIS price for drugs on the essential medicines list, and assuming a daily dose as recommended in the Ghana Standard Treatment Guidelines (median of range) [22, 25, 37]. Some data were available on the level of prescribing by drug, but not by brand or formulation. Based on the NHIS price, and estimated use within class, the mean cost per year ranged from GH₵ 26 per year for diuretics to GH₵ 399 per year for CCBs (see Table 12). In sensitivity analysis, we also tested the impact of using the least and most expensive drug and formulation within each class.

	Daily dose	Cost per year NHIS	Lowest generic	Highest branded	% use in class NHIS
ACEi					
**Lisinopril 2.5mg Tablet**	22.5	690	690	690	25%
**Lisinopril 5mg Tablet**	22.5	345	181	2957	25%
**Lisinopril 10mg Tablet**	22.5	246	205	2751	25%
**Lisinopril 20mg Tablet**	22.5	177	90	1992	25%
**Ramipril 2.5mg Tablet**	6.3	274	256	420	0%
**Ramipril 5 mg Tablet**	6.3	228	160	228	0%
					364
ARB					
**Losartan 25mg Tablet**	62.5	365	237	365	33%
**Losartan 50mg Tablet**	62.5	228	125	282	33%
**Losartan 100mg Tablet**	62.5	183	91	237	33%
					259
BETA BLOCKERS					
**Atenolol 25 mg Tablet**	75	142	126	142	33%
**Atenolol 50 mg Tablet**	75	110	77	110	33%
**Atenolol 100 mg Tablet**	75	55	49	55	33%
					102
CCB					
**Amlodipine 5mg Tablet**	7.5	110	93	2738	8%
**Amlodipine 10mg Tablet**	7.5	82	52	2190	8%
**Nifedipine 10mg Capsules**	50	894	894	1898	21%
**Nifedipine 10mg (slow release)**	50	456	456	548	21%
**Nifedipine 30mg (GITS)**	50	335	335	1570	21%
**Nifedipine 20mg (slow release)**	50	155	155	456	21%
					399
DIURETICS					
**Bendrofluazide 2.5 mg Tablet**	2.5	26	26	51	100%
**Bendrofluazide 5 mg Tablet**	2.5	18	18	26	
					26

Table 12 Antihypertensive prices

** Ettehad et al (2016)[4] for CHD, stoke, heart failure and all cause mortality.*

Elliot and Meyer (2007) [5] for incident type 2 diabetes

Adverse event costs

The unit costs of services were based on a weighted average of NHIS tariffs for public hospitals, private hospitals and tertiary hospitals (Table 13) [13-15]. For the base case analysis we assumed a distribution of 40%, 40% and 20% for public, private and tertiary hospitals respectively, with uncertainty over this distribution included in the PSA (Dirichlet distribution, with assumed sample size of 100 to reflect high uncertainty over this assumption).

	G-DRG	Public	Private	Tertiary	Value us
MDC: MEDICAL					
**DRG - Ischaemic Heart Disease**	MEDI33A	175.82	245.53	225.56	213
**DRG - CVA/ stroke**	MEDI14A	205.71	298.84	315.88	265
**DRG - Heart disease**	MEDI07A	220.16	333.35	324.47	286
**DRG - Diabetes (simple >12 years)**	MEDI02A	154.40	240.64	197.25	197
MDC: OUTPATIENT					
**General OPD - Adult**	OPDC06A	11.42	19.11	19.97	16
MDC: INVESTIGATIONS					
**ECG**	INVE41D	4.49	6.21	5.33	5
**Full blood count (FBC automation)**	INVE52D	6.24	6.76	7.08	6
**Liver function test (LFT)**	INVE74D	11.42	11.83	12.28	11
**Lipid profile**	INVE75D	10.34	10.86	11.20	10
**Renal function tests (U&Es)**	INVE21D	7.62	8.13	8.47	7
**2 hour post prandial blood glucose**	INVE01D	5.51	6.56	6.35	6
**Fasting blood sugar/ random blood sugar**	INVE46D	3.29	3.56	4.14	3
COMMUNITY					
**Physician visit (GP)**					10
**Pharmacist (per day)**					100
**Pharmacy assistant (per day)**					50

Table 13 Unit costs of services: Ghana NHIS Tariffs [13-15]

Estimates of the cost to the NHIS of diagnosis, treatment and care associated with adverse events are shown in Table 14 (year 1) and Table 15 (subsequent years).
The percentage of patients assumed to access a package of covered services was 80% in the base case, varying between 60% and 100% (uniform distribution) in PSA. The package of services for stroke was based on recommended outpatient follow up every 2 weeks for 4 times after discharge, then every month for 3 times, then every six months for at least 3 years. We assumed similar follow up after acute admission for CHD and heart failure.

	% patients	Quantity	Unit cost	Cost
**Coronary Heart Disease**				
**DRG - Acute admission**	0.80	1	213.65	170.92
**Follow-up specialist consultation**	0.80	7	16.21	90.75
**ECG**	0.80	7	5.35	29.94
**Blood tests (FBC, Lipids, PPBG, LFTs)**	0.80	7	35.19	197.06
**Drugs (Asp, BB, ACEi, Statin)**	0.80	1	30.00	24.00
				512.68
**Stroke**				
**DRG - Acute admission**	0.80	1	265.00	212.00
**Follow-up specialist consultation**	0.80	7	16.21	90.75
**Blood tests (FBC, Lipids, PPBG, LFTs)**	0.80	7	35.19	197.06
**Drugs (Asp or warfarin, anti-BP, Statin)**	0.80	1	34.00	27.20
				527.01
**Heart failure**				
**DRG acute admission**	0.80	1	286.30	229.04
**Consultations (specialist)**	0.80	7	16.21	90.75
**ECG**	0.80	7	5.35	29.94
**Blood tests (FBC, Lipids, PPBG, LFTs)**	0.80	7	35.19	197.06
**Drugs (ACEi, BB, Diuretic, Dig, Spiro)**	0.80	1	22.00	17.60
				564.39
**Diabetes**				
**DRG - Diabetes**	0.80	1	197.47	157.97
**Consultations (specialist)**	0.80	1	16.21	12.96
**ECG**	0.80	1	5.35	4.28
**Blood tests (PPBG, Lipids, LFT, RFT)**	0.80	1	36.57	29.25
**Physician visit (GP)**	0.80	2	10.20	16.32
**Fasting blood glucose**	0.80	6	3.57	17.13
**Annual eye and feet tests**	0.80	1	16.21	12.96
**Drugs (Hypog, Asp, ACEi, Statin)**	0.80	1	29.00	23.20
				274.08

Table 14 Cost of adverse events: first year

	% patients	Quantity	Unit cost	Cost
**Coronary Heart Disease**				
Specialist visit	0.80	2	16.21	25.93
ECG	0.80	2	5.35	8.55
Blood tests (FBC, Lipids, PPBG, LFTs)	0.80	2	35.19	56.30
Drugs (Asp, BB, ACEi, Statin)	0.80	1	30.00	24.00
				114.79
**Stroke**				
Stroke				
Specialist visit	0.80	2	16.21	25.93
Blood tests (FBC, Lipids, PPBG, LFTs)	0.80	2	35.19	56.30
Drugs (Asp or warfarin, anti-BP, Statin)	0.80	1	34.00	27.20
				109.43
**Heart failure**				
Heart failure				
Specialist visit	0.80	2	16.21	25.93
ECG	0.80	2	5.35	8.55
Blood tests (FBC, Lipids, PPBG, LFTs)	0.80	2	35.19	56.30
Drugs (ACEi, BB, Diuretic, Dig, Spiro)	0.80	1	22.00	17.60
				108.39
**Diabetes**				
Diabetes				
Physician visit (GP)	0.80	2	10.20	16.32
Blood tests (PPBG, Lipids, LFT, RFT)	0.80	2	36.57	58.51
Fasting blood glucose	0.80	2	3.57	5.71
Annual Eye and feet tests	0.80	1	19.11	15.29
Drugs (Hypog, Asp, ACEi, Statin)	0.80	1	29.00	23.20
				119.03

Table 15 Cost of adverse events: subsequent year

DALY calculations

Years of life lost by age, and the disability loss per year lived with CHD, stroke, heart failure and type 2 diabetes were calculated. Years of life lost by age are based on standard life expectancy as set by WHO Global Health Estimates [16], discounted at 3% per year. Please see Table 16 for WHO Standard Life Table for Years of Life Lost (YLL).

Disability weights for CHD, stroke, heart failure and type 2 diabetes were 0.124, 0.266, 0.201 and 0.015 respectively, from the 2003 WHO estimates [17]. We could not use more recent estimates of disability weights, because these have not been presented as averages for these broad conditions, but by a more detailed breakdown of level of disability and type of complication [18]. In the base case, the model uses the default constant of 0.1658. In accordance with the WHO adopted simplified DALYs calculations there was no discounting for age-weight in our DALY calculations [16]. These parameters can be changed for sensitivity analysis.

Age	SEYLL*	Age	SEYLL	Age	SEYLL
0	91.94	35	57.15	70	23.15
1	91.00	36	56.16	71	22.23
2	90.01	37	55.17	72	21.31
3	89.01	38	54.18	73	20.40
4	88.02	39	53.19	74	19.51
5	87.02	40	52.20	75	18.62
6	86.02	41	51.21	76	17.75
7	85.02	42	50.22	77	16.89
8	84.02	43	49.24	78	16.05
9	83.03	44	48.25	79	15.22
10	82.03	45	47.27	80	14.41
11	81.03	46	46.28	81	13.63
12	80.03	47	45.30	82	12.86
13	79.03	48	44.32	83	12.11
14	78.04	49	43.34	84	11.39
15	77.04	50	42.36	85	10.70
16	76.04	51	41.38	86	10.03
17	75.04	52	40.41	87	9.38
18	74.05	53	39.43	88	8.76
19	73.05	54	38.46	89	8.16
20	72.06	55	37.49	90	7.60
21	71.06	56	36.52	91	7.06
22	70.07	57	35.55	92	6.55
23	69.07	58	34.58	93	6.07
24	68.08	59	33.62	94	5.60
25	67.08	60	32.65	95	5.13
26	66.09	61	31.69	96	4.65
27	65.09	62	30.73	97	4.18
28	64.10	63	29.77	98	3.70
29	63.11	64	28.82	99	3.24
30	62.11	65	27.86	100	2.79
31	61.12	66	26.91	101	2.36
32	60.13	67	25.96	102	1.94
33	59.13	68	25.02	103	1.59
34	58.14	69	24.08	104	1.28
				105	1.02

*SEYLL: standard expected years of life lost. Based on projected frontier period life expectancy and life table for year 2050 (UN Population Division 2013).

Table 16 WHO Standard Life Table for Years of Life Lost (YLL) [16]

viii. Cost effectiveness acceptability curve

Figure 2 below shows the cost-effectiveness acceptability curves for the most cost-effective options. Below a willingness to pay threshold of GH₵ 200 per DALY avoided, the probability that any antihypertensive treatment is cost effective is negligible. Between a threshold of

about GH₵ 400 and 8,600 per DALY avoided, it appears almost certain that diuretics are the most cost-effective option. Above GH₵ 8,600 cedi per DALY avoided (about twice Gross National Income (GNI) per capita) the probability that CCB are cost-effective begins to rise, reaching 100% of simulations at GH₵ 15,100 cedi per DALY avoided.

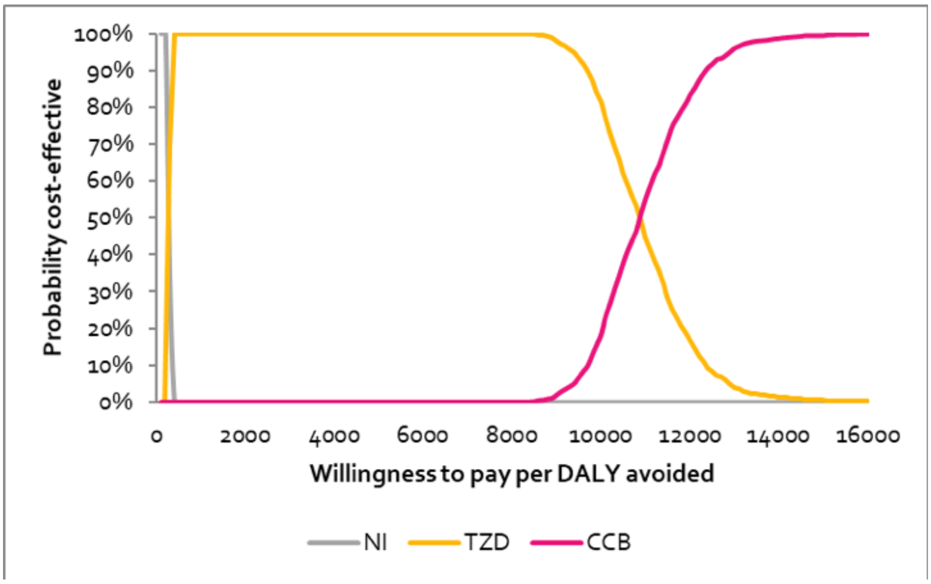

Figure 2 Cost-effectiveness acceptability curve (CEAC)

Section B – Discussion

i. Comparing this Study's ICER value with other studies in the Ghanaian context

The estimates presented here for the most cost-effective hypertension treatment options are in line with results of other analyses undertaken within a Ghanaian context, looking at different technologies in other disease areas. In the Zelle et al (2012) study for example, which looked at the cost effectiveness of various breast cancer control options, it would seem that thiazides and CCBs are at least as cost-effective as screening by clinical breast examination or undertaking an awareness raising campaign using mass media, and substantially more cost-effective than mammography screening of women of aged 40–69 years.

After adjusting for inflation from year 2009 to 2017, the ICER values from Zelle et al study for breast cancer control interventions shows that the most cost-effective intervention (CBE and treatment of all stages breast cancer) as highlighted by the study, has an ICER value pf $3,340/DALY averted comparable to $2,456/ DALY averted for CCBs use in management of hypertension. Both studies used modelling and adaptation techniques undertaken by identifying data sources for the collection of country-specific data, and their analyses were based on local demographic, epidemiological and economic data, to the extent these data were available within the Ghanaian context. See Table 8.

Inflation adjustment	
Consumer Price Index of 2017 (Ghana)	232.256
Consumer Price Index of 2009 (Ghana)	90.328

Study details			Referenced value		Adjustments	
Study	Referenced year	Intervention	ICER (GH₵ per DALY averted)	ICER value ($ per DALY averted)	Inflation factor	Adjusted ICER Value (2017)
**Ghana Hypertension Study**	2017	Thaizides	276	61	-	**61**
		Calcium Channel Blockers (CCBs)	11,061	2,456	-	**2,456**
Zelle et al. 2012	2009	Biennial screening by clinical breast examination (CBE) of women aged 40-69 years, in combination with treatment of all stages	1,853	1,299	2.57	**3,340**
		Mass media awareness raising	1,946	1,364	2.57	**3,505**
		Mammography screening of women of aged 40–69 years	18,414	12,908	2.57	**33,174**

Table 8 Comparison of different ICER values across various interventions in Ghanaian Context

ii. Pricing and procurement

The cost-effectiveness results were highly sensitive to assumptions about the price of drug formulations. For example, if the lowest price CCB is used (generic amlodipine 10mg tablet, 52 GH₵ per year), rather than the NHIS median (399 GH₵ per year), the incremental cost effectiveness ratio (ICER) for CCB vs. diuretic falls to GH₵ 806 per DALY avoided. Conversely, the most expensive CCB covered by the NHIS (branded amlodipine 5mg, at 2,738 GH₵ per year) has an ICER of over GH₵ 80,231 per DALY avoided. Similar estimates have been calculated for thiazides (See Table 9 below). This highlights the importance of implementing more effective mechanisms aimed at pricing and procurement. The analysis suggests there are significant potential savings by switching to lower priced formulations or negotiating lower prices for medicines. This generates potential savings in budget impact represented by 83% drop in budget impact value for CCBs and 35% for thiazides.

Class	Scenario	Drug formulation	**Cost** (GH₵ per patient per year)	**ICER compared to no intervention** (GH₵ per DALY avoided)	**% change from default ICER value**	**5-year Budget Impact** (GH₵ million)	**% change from default Budget Impact value**
**Calcium Channel Blockers**	**Default**	Weighted average based on current NHIS formulations use across brands	**399**	**11,061**	n/a	**510**	n/a
	Highest price formulation	Branded Amlodipine 5 mg	2,738	80,231	625%	3,525	591%
	Lowest price formulation	Generic Amlodipine 10 mg	52	806	-93%	87	-83%
**Thiazides**	**Default**	Weighted average based on current NHIS formulation use across brands	**25.6**	**276**	n/a	**26**	n/a
	Highest price formulation	Bendrofluazide 2.5 mg Tablet	51	606	120%	59	127%
	Lowest price formulation	Bendrofluazide 5 mg Tablet	18	183	-34%	17	-35%

Table 9 Effect of drug prices on ICER and budget impact

Section C- iDSI reference case assessment of Ghana hypertension model

	Statement of principle	Reference Case Methods Specifications	Ghana Hypertension Model Specifications	Comments
1	**An economic evaluation should be communicated clearly and transparently to allow the decision maker(s) to interpret the methods and results**	• The decision problem must be fully and accurately described and the economic evaluation characterised	✓	
		• Limitations of the economic evaluation in informing policy should be characterised	✓	
		• Declarations of interest should be made	✓	
2	**The comparators against which costs and effects are measured should accurately reflect the decision problem.**	• At a minimum, the following comparative analysis should be undertaken:<br>○ The intervention(s) that is(are) currently offered to the population as defined in the decision problem as the base case comparator<br>○ A “do nothing” analysis representing best supportive (non-interventional care) for the population as additional analysis	✓	

3	**An economic evaluation should consider all available evidence relevant to the decision problem**	• A systematic and transparent approach should be taken to obtain evidence and make judgements about evidence exclusion	✓	
		• Estimates of clinical effect of intervention and comparator(s) should be informed by a systematic review of the literature	✓	
		• Single-study or trial-based analyses should outline how the single study or trial is a sufficient source of evidence and should ensure that the stated decision problem is specific to particular context and time of the study or trial	✓	
		• Budget and time allocated to perform an economic evaluation should not determine selection of evidence	✓	
4	**The measure of health outcome should be appropriate to the decision problem, should capture positive and negative effects on length of life and quality of life, and should be generalizable across disease states**	• Disability-Adjusted Life Years (DALYs) averted should be used [stated methodological specification]	✓	
		• Other generic measures that capture length and quality of life (e.g. the QALY) can be used in separate analysis where information is available	✓	

5	**All differences between the intervention and the comparator in expected resource use and costs of delivery to the target population(s) should be incorporated into the evaluation**	• Estimates should reflect the resource use and unit costs/prices that are expected if the intervention were to be rolled out to the population defined in the decision problem	✓	
		• Costs not included in study settings used to inform the analysis but would be incurred if the intervention was rolled out should be included in the base case analysis	✓	
		• All resource implications relevant to the decision problem should be costed, including donated inputs and out of pocket inputs from individuals	✗	This version of the model only includes costs to the NHIS. It was agreed that further extensions of this model can explore out of pocket inputs.
		• Analysis should include estimation of changes in costs estimates due to scalability	✓	
6	**The time horizon used in an economic evaluation should**	• Lifetime time horizon should be used in first instance	✓	

	**be of sufficient length to capture all costs and effects relevant to the decision problem; an appropriate discount rate should be used to discount cost and effects to present value**	• Shorter time horizon can be used where shown that all costs and effects that are relevant to the decision problem have been captured	✓	
		• A 3% annual discount rate for costs and effects should be used in base case analysis [stated methodological specification]. Additional analysis exploring differing discount rates appropriate to the decision problem should be used	✓	
		• Additional analysis should explore an annual discount rate that reflects the rate at which the government can borrow funds on the international market	✗	It is straightforward to conduct sensitivity analysis on the discount rates, but advice is needed on government borrowing rates.
		• Where time horizon used is greater than 30 years, the impact of lower discount rates should be explored in sensitivity analysis	n/a	
**7**	**Non-health effects and costs associated with gaining or providing access to health interventions that don't**	• Base case analysis should reflect direct health costs and health outcomes; however the analysis	✓	

	**accrue to the health budget should be identified where relevant to the decision problem. All costs and effects should be disaggregated, either by sector of the economy or to whom they accrue**	should adopt a disaggregated societal perspective		
		• Non-health effects and costs that fall outside the health budget should be included in additional analysis; the mechanism of inclusion will differ depending on the decision problem and context	✗	It was agreed to potentially consider this in future extensions of the model.
		• Where external funding or individual OOP payments substitute for costs that would otherwise fall on a health budget, these costs should be included in the base case analysis, however the impact of excluding these payments must be explored in sensitivity analysis	✗	This version of the model only includes costs to the NHIS. It was agreed that further extensions of this model can explore out of pocket inputs
**8**	**The cost and effects of the intervention on sub-populations within the decision problem should be explored and the implications appropriately characterised**	• Heterogeneity should be explored in subgroups of the population identified in the decision problem, where subgroup formation should be informed by:<br>○ Relevant effect of the intervention differs in different populations<br>○ Characteristics of different populations that may influence the absolute health effects<br>○ Characteristics that influence direct costs of provision or other associated costs such	✓	

		as geographical location across the constituency		
		• Subgroup analysis should always be determined by:<br>○ The evidence base regarding differences in relative effect, baseline risk or other characteristics<br>○ Whether the differences are likely to have an important influence on costs and effects	✓	
9	**The uncertainty associated with an economic evaluation should be appropriately characterised**	• The economic evaluation should explore:<br>○ Uncertainty in the structure of the analysis<br>○ Uncertainty due to source of parameters<br>○ Uncertainty due to precision of parameters	✓	
10	**The impact of implementing the intervention on the health budget and on other constraints should be identified clearly and separately**	• Budget impact analysis should be performed that provides an estimate of the implications of implementing the intervention on various budgets	✓	
		• Budget impact analysis should reflect the decision problem and the constituency in which the intervention will be implemented	✓	

**11**	**An economic evaluation should explore the equity implications of implementing the intervention**	• There are various mechanisms available for how the equity implications of an intervention should be assessed. The method chosen should be appropriate to the decision problem and justifiable to the decision-maker	✘	There was discussion of whether to disaggregate results for sections of the population (e.g. urban vs. rural). However, policy makers wanted to focus initially on national average results.
		• Equity implications should be considered at all stages of the economic evaluation, including design, analysis and reporting	✘	As above.

Section D – Acknowledgements

Below is the full list of the HTA Technical Working group members:

Name & position	Affiliation
Martha Gyansa-Lutterodt,	MoH Director of Pharmaceutical Services
Edith Gavor	PM, GNDP, MoH (Deputy Director of Pharmaceutical Services)
Brian Asare	Programme Officer, GNDP, MoH
Saviour Yevutse	MoH, Directorate of Pharmaceutical Services
Dr Koku Awoonor-Williams, Director, PPMED	Ghana Health Service Institutional Care Division,
Dr Cynthia Bannerman	Deputy Director, Institutional Care Division, quality assurance; GHS
Dr Justice Nonvignon	Senior Lecturer, School of Public Health, College of Health Sciences, University of Ghana
Prof Charles Ansah	Dean, FPPS, KNUST, Kumasi
Prof Francis Ofei	Dean, SMS, UCC and Chair of the Ghana STG
Dr. Marc Dzradosi	STG Review Team
Dr Lydia Dsane-Selby	NHIA
Dr Memuna Tanko	NHIA
Cecilia Senoo	Coalition of NGOs in Health
Dr Emmanuel Odame	MOH Policy Planning Monitoring and Evaluation (PPME) Directorate
Prof Alex Dodoo	College of Health Sciences, UG
Edith Annan	WHO Ghana Office
Ruby Awittor	Ghana Health Service. Ashiama District
Jennifer Manfo	GHS, Ashanti Regional Health Directorate
Dr Adwoa -Benneh	NHIA
Dr Berko Panyin Anto	FPPS, KNUST
George Hedidor	National Drug Resource Information Centre (NDIRC), MOH

References

1. National Collaborating Centre for Chronic Conditions (UK). *Hypertension: Management in Adults in Primary Care: Pharmacological Update. (NICE Clinical Guidelines, No. 34.)*. 2006 03.07.2017]; Available from: https://www.ncbi.nlm.nih.gov/books/NBK45886/.
2. Marfo, A.F. and F.T. Owusu-Daaku, *Evaluation of a pharmacist-led hypertension preventative and detection service in the Ghanaian community pharmacy: an exploratory study.* Int J Pharm Pract, 2016. **24**(5): p. 341-8.
3. Johnson, J.A., *Ethnic differences in cardiovascular drug response: potential contribution of pharmacogenetics.* Circulation, 2008. **118**(13): p. 1383-93.
4. Ettehad, D., et al., *Blood pressure lowering for prevention of cardiovascular disease and death: a systematic review and meta-analysis.* The Lancet, 2016. **387**(10022): p. 957-967.
5. Elliott, W.J. and P.M. Meyer, *Incident diabetes in clinical trials of antihypertensive drugs: a network meta-analysis.* The Lancet, 2007. **369**(9557): p. 201-207.
6. Brewster, L.M., G.A. van Montfrans, and J. Kleijnen, *Systematic review: Antihypertensive drug therapy in black patients.* Annals of Internal Medicine, 2004. **141**(8): p. 614-627.
7. Ghana Statistical Service. *Population and Housing Census 2010*. 2012; Available from: http://www.statsghana.gov.gh/docfiles/2010phc/Census2010_Summary_report_of_final_results.pdf.
8. Ghana Statistical Service (GSS), Ghana Health Service (GHS), and ICF International, *Ghana Demographic and Health Survey 2014 (GDHS)*. 2015: Rockville, Maryland, USA.
9. World Health Organization (WHO). *Life tables by country- Ghana*. 2016 09.05.2017]; Available from: http://apps.who.int/gho/data/?theme=main&vid=60630.
10. Hippisley-Cox, J., et al., *Predicting cardiovascular risk in England and Wales: prospective derivation and validation of QRISK2.* British medical journal, 2008. **336**(7659): p. 1475-1482.
11. Singh, G.M., et al., *The age-specific quantitative effects of metabolic risk factors on cardiovascular diseases and diabetes: a pooled analysis.* PLoS One, 2013. **8**(7): p. e65174.
12. Hippisley-Cox, J., et al., *Predicting risk of type 2 diabetes in England and Wales: prospective derivation and validation of QDScore.* Bmj, 2009. **338**: p. b880.
13. National Health Insurance Authority (NHIA), *National Health Insurance Scheme Tariffs for secondary care hospital - private*. 2013: Accra, Ghana.
14. National Health Insurance Authority (NHIA), *National Health Insurance Scheme Tariffs for secondary care hospital - public*. 2013: Accra, Ghana.
15. National Health Insurance Authority (NHIA), *National Health Insurance Scheme Tariffs for tertiary hospitals*. 2013: Accra, Ghana.
16. World Health Organisation (WHO). *WHO methods and data sources for global burden of disease estimates 2000-2015*. 2017 [cited 2018 20 November 2018]; Available from:

http://www.who.int/healthinfo/global_burden_disease/GlobalDALYmethods_2000_2015.pdf?ua=1.
17. World Health Organisation (WHO). *Global Burden of Disease 2004 update: Disability weights for diseases and conditions*. 2004; Available from: https://www.who.int/healthinfo/global_burden_disease/GBD2004_DisabilityWeights.pdf.
18. Salomon, J.A., et al., *Disability weights for the Global Burden of Disease 2013 study.* The Lancet Global Health, 2015. **3**(11): p. e712-e723.
