# Supplementary material for: Supporting the Development of Evidence-Informed Policy Options: An Economic Evaluation of Hypertension Management in Ghana
Source: Value Health. 2020 Feb;23(2):171–9. doi: 10.1016/j.jval.2019.09.2749 (PMC7065042; doi:10.1016/j.jval.2019.09.2749)
Supplement: Supplementary Material 2 [file mmc2.docx]

**Supplementary Material** – Supporting the development of evidence-informed coverage decisions: an economic evaluation of hypertension management options in Ghana

# Introduction

This appendix describes Version 3.0 of the Ghana Hypertension Model. The model was programmed in Excel by Joanne Lord, initially based on a model developed for the 2006 update of the NICE clinical guideline on hypertension [1], and adapted for Ghana following discussion and agreement of appropriate modelling assumptions and data inputs with the Technical Working Group (see part C) and Kalipso Chalkidou, Francoise Cluzeau and Mohamed Gad. The model is available under a Creative Commons Attribution-Non Commercial-Share Alike license (CC BY-NC-SA 4.0).

# **Section A – The Model**

## i. Aims and objectives

The aim of the model is to provide a platform to estimate the cost-effectiveness and budget impact of a range of interventions to prevent cardiovascular disease (CVD) through better control of hypertension in the Ghanaian population.

## ii. Population and subgroups

The model estimates outcomes for patients with essential hypertension, excluding those with pre-existing CVD, diabetes and pregnant women. 48 subgroups of prevalent cases are defined by: men and women; by 6 ten-year age groups (from 20-29 to 70+); and 4 levels of blood pressure (normal due to effective control with antihypertensive medication, mild, moderate or severe; see Table 1). In addition, patients with raised blood pressure are further divided according to current treatment status (not aware of raised blood pressure; aware but not treated; treated but not controlled). Thus, in total the model includes 120 subgroups of patients (see Table 2).

Table 1 Classification of hypertension by severity

## iii. Intervention

The model can be used to compare expected outcomes assuming no treatment or monotherapy with one of the 5 main classes of antihypertensive medication:

1. Angiotensin Converting Enzyme Inhibitor (ACEi)
2. Angiotensin Receptor Blockers (ARB)
3. Betablockers (BB)
4. Calcium Channel Blockers (CCB)
5. Thiazide like diuretics (TZD)

## iv. Policy Scenarios

The analyses presented in this report include the following types of policy change:

1. Price reductions for listed drugs: e.g. 10% reduction in mean price of ACEi, ARB, BB and CCB, no change in numbers of patients treated by drug class.
2. Reduced use of less effective/more expensive drugs: e.g. switch 10% of patients currently treated with ACEi, ARB and BB to TZD.
3. Increased use of better value drugs: e.g. switch 10% of patients treated with CCB to TZD.
4. Increased treatment of diagnosed patients: e.g. use of TZD for patients aware of hypertension but not currently on treatment.
5. Increased case detection: e.g. 5% of population aged 40 years and over offered screening in community pharmacy [2].

For simplicity, the policy options that were modelled assumed a one-off change, instituted in a single year for a proportion of the current prevalent population with hypertension. The model then estimates consequent health effects and costs over the remaining lifetime for those individuals. The impact of extending the policies to more patients in future years (whether members of the initial prevalent population or to new incident cases) could be estimated in separate modelled scenarios.

Note that the modelled examples do not generally assume 100% uptake of the suggested treatment changes, because it is not likely that such major shifts in practice could be achieved in a single year. In addition, there are individual factors not included in the model that mean that the changes would not be clinically appropriate for all patients. For example, treatment may reasonably differ for patients with co- morbidities: such as use of beta blockers for patients with cardiac arrhythmia or ACEi/ARB for those with heart failure. And some patients may be contra-indicated or intolerant of some antihypertensive medications.

It should also be noted that the estimated numbers of people in the modelled subgroups include people with secondary hypertension (attributable to a specific secondary cause) and also some people with pre-existing diabetes and/or cardiovascular disease. The model estimates of baseline risk and treatment effectiveness will not be accurate for these groups.

## v. Outcomes

The model estimates the incidence of five types of adverse event related to hypertension and modified by the use of antihypertensive medication:

- Coronary Heart Disease (CHD)
- Stroke
- Heart failure
- Type 2 diabetes and
- All-cause mortality.

In addition, Disability Adjusted Life Years (DALYs) and healthcare costs associated with interventions and incident adverse events are estimated.

## vi. Parameters and sources of data

**Population**

The number of people in the Ghanaian population by sex and included age groups (age 20 years and older) was estimated from the 2010 census (See Table 2) [3]. People under the age of 20 were not included in the model, as we assumed that the prevalence of essential hypertension in children and teenagers would be negligible.

Table 2 Number of people by sex and age group (2010 Census)

The population in Ghana has grown since 2010, but for simplicity in this version of the model, the numbers have not been inflated to reflect this. we also present result for the whole nation, but it would be possible to estimate costs and effects by subgroup.

**Prevalence of hypertension**

The 2014 Ghana DHS included measurement of blood pressure for a representative sample of the population: including 9,396 women between the ages of 15 and 49 and 4,388 men aged 15 to 59 [4]. These individuals were also asked if they were aware of having high blood pressure and if they were receiving treatment for high blood pressure.

The proportions of men and women in each age group with controlled, mild, moderate, and severe hypertension are shown in Figure 1. A greater proportion of women with hypertension were aware of this condition than were men with hypertension. As might be expected, a greater proportion of older people had hypertension than did young people (with a steeper percentage increase per year in women).

Figure 1 Prevalence of Hypertension (GDHS 2014)

The numbers of people in the 120-modelled subgroups were estimated from the data in Table 3. The prevalence of hypertension in men over the age of 60 and women over the age of 50 were estimated by simple linear extrapolation from the observed prevalence in younger age groups: 0.79% (standard error (SE): 0.05%) increase per year for men and 1.14% per year for women (SE 0.15%). The numbers of people in each subgroup were sampled probabilistically for the PSA: using beta distributions to sample the prevalence of hypertension and Dirichlet distributions to sample the split of those with controlled, mild, moderate and severe hypertension for each age/sex group.

Table 3 Estimated number of people with hypertension by subgroup

**Baseline risks of adverse events**

Annual mortality rates for individuals without CVD, diabetes or heart failure were taken from the World Health Organization life table for Ghana, published in 2016 (See Table 4) [5].

Table 4 All-cause mortality with no prior event (WHO Ghana life table 2016)

The annual probabilities of first incidence of CHD, stroke, heart failure and diabetes for each subgroup in the absence of treatment were estimated from international data. It would have been preferable to use estimates of incidence from Ghana, or other West African countries with a similar population and healthcare profile, but we have not been able to identify good quality cohort studies with longitudinal follow up of a population sample from these contexts.

Baseline estimates of the incidence of CVD (CHD and stroke) were taken from a multivariate analysis of primary care data for black African patients living in the UK (the QRisk2 algorithm) [6]. The derivation cohort included 6,917 individuals of Black African ethnicity, with 33 incidence CVD cases observed in 12,869-person years of follow up. The resulting estimates of incidence over ten years for men and women by age group in the absence of other risk factors are shown in Table 5. For the PSA, incidences by age/sex subgroup were sampled from beta distributions. Additional uncertainty was assumed around these probabilistic estimates due to the extrapolation of UK data (by assuming an effective sample size of 200 per subgroup).

Table 5 Ten-year Incidence of CVD in black African patients with no other risk factors (QRisk2)

The relative incidences of CHD, stroke and heart failure were estimated from a meta-analysis of trials of antihypertensive medications [7]. This reported 3,928, 2,220 and 1,844 incident cases of CHD, stroke and heart failure over a total 205,828-person years of follow up: proportions of coronary events and heart failure per case of CVD were 36.8% and 38.3% respectively. These proportions were sampled for the PSA using beta distributions.

The QRisk2 estimates of incidence cited in Table 6 are for people with normal blood pressure (SBP of 135) and no other risk factors. We adjusted the estimated risks of CHD, stroke and heart failure for people with mild, moderate and severe hypertension using relative risks per 10mm Hg increase in SBP from a pooled analysis of large cohort studies (see Table 6)[8]. These relative risks were sampled probabilistically from a log-normal distribution, with standard error based on the reported 95% confidence intervals.

Table 6 Relative risk per 10mm Hg higher SBP (Singh et al 2013)

# **Section B – Discussion**

## i. Comparing this Study’s ICER value with other studies in the Ghanaian context

The estimates presented here for the most cost-effective hypertension treatment options are in line with results of other analyses undertaken within a Ghanaian context, looking at different technologies in other disease areas. In the Zelle et al (2012) study for example, which looked at the cost effectiveness of various breast cancer control options, it would seem that thiazides and CCBs are at least as cost-effective as screening by clinical breast examination or undertaking an awareness raising campaign using mass media, and substantially more cost-effective than mammography screening of women of aged 40–69 years.

After adjusting for inflation from year 2009 to 2017, the ICER values from Zelle et al study for breast cancer control interventions shows that the most cost-effective intervention (CBE and treatment of all stages breast cancer) as highlighted by the study, has an ICER value pf $3,340/DALY averted comparable to $2,456/ DALY averted for CCBs use in management of hypertension. Both studies used modelling and adaptation techniques undertaken by identifying data sources for the collection of country-specific data, and their analyses were based on local demographic, epidemiological and economic data, to the extent these data were available within the Ghanaian context. See Table 7.

| **Inflation adjustment** |  |
| --- | --- |
| Consumer Price Index of 2017 (Ghana) | 232.256 |
| Consumer Price Index of 2009 (Ghana) | 90.328 |

| **Study details** | | | **Referenced value** | | **Adjustments** | |
| --- | --- | --- | --- | --- | --- | --- |
| **Study** | **Referenced year** | **Intervention** | **ICER  (GH₵ per DALY averted)** | **ICER value  ($ per DALY averted)** | **Inflation factor** | **Adjusted ICER Value (2017)** |
| **Ghana Hypertension Study** | 2017 | Thaizides | 276 | 61 | - | **61** |
|  |  | Calcium Channel Blockers (CCBs) | 11,061 | 2,456 | - | **2,456** |
| Zelle et al.  2012 | 2009 | Biennial screening by clinical breast examination (CBE) of women aged 40-69 years, in combination with treatment of all stages | 1,853 | 1,299 | 2.57 | **3,340** |
|  |  | Mass media awareness raising | 1,946 | 1,364 | 2.57 | **3,505** |
|  |  | Mammography screening of women of aged 40–69 years | 18,414 | 12,908 | 2.57 | **33,174** |

Table 7 Comparison of different ICER values across various interventions in Ghanaian Context

## ii. Pricing and procurement

The cost-effectiveness results were highly sensitive to assumptions about the price of drug formulations. For example, if the lowest price CCB is used (generic amlodipine 10mg tablet, 52 GH₵ per year), rather than the NHIS median (399 GH₵ per year), the incremental cost effectiveness ratio (ICER) for CCB vs. diuretic falls to GH₵ 806 per DALY avoided. Conversely, the most expensive CCB covered by the NHIS (branded amlodipine 5mg, at 2,738 GH₵ per year) has an ICER of over GH₵ 80,231 per DALY avoided. Similar estimates have been calculated for thiazides (See Table 8 below). This highlights the importance of implementing more effective mechanisms aimed at pricing and procurement. The analysis suggests there are significant potential savings by switching to lower priced formulations or negotiating lower prices for medicines. This generates potential savings in budget impact represented by 83% drop in budget impact value for CCBs and 35% for thiazides.

| **Class** | **Scenario** | **Drug formulation** | **Cost**  (GH₵ per patient per year) | **ICER compared to no intervention** (GH₵ per DALY avoided) | **% change from default ICER value** | **5-year Budget Impact** (GH₵ million) | **% change from default Budget Impact value** |
| --- | --- | --- | --- | --- | --- | --- | --- |
| **Calcium Channel Blockers** | **Default** | Weighted average based on current NHIS formulations use across brands | **399** | **11,061** | n/a | **510** | n/a |
|  | Highest price formulation | Branded Amlodipine 5 mg | 2,738 | 80,231 | 625% | 3,525 | 591% |
|  | Lowest price formulation | Generic Amlodipine 10 mg | 52 | 806 | -93% | 87 | -83% |
| **Thiazides** | **Default** | Weighted average based on current NHIS formulation use across brands | **25.6** | **276** | n/a | **26** | n/a |
|  | Highest price formulation | Bendrofluazide 2.5 mg Tablet | 51 | 606 | 120% | 59 | 127% |
|  | Lowest price formulation | Bendrofluazide 5 mg Tablet | 18 | 183 | -34% | 17 | -35% |

Table 8 Effect of drug prices on ICER and budget impact

# **Section C – Acknowledgements**

Below is the full list of the HTA Technical Working group members:

| **Name & position** | **Affiliation** |
| --- | --- |
| Martha Gyansa-Lutterodt, | MoH Director of Pharmaceutical Services |
| Edith Gavor | PM, GNDP, MoH (Deputy Director of Pharmaceutical Services) |
| Brian Asare | Programme Officer, GNDP, MoH |
| Saviour Yevutse | MoH, Directorate of Pharmaceutical Services |
| Dr Koku Awoonor-Williams, Director, PPMED | Ghana Health Service Institutional Care Division, |
| Dr Cynthia Bannerman | Deputy Director, Institutional Care Division, quality assurance; GHS |
| Dr Justice Nonvignon | Senior Lecturer, School of Public Health, College of Health Sciences, University of Ghana |
| Prof Charles Ansah | Dean, FPPS, KNUST, Kumasi |
| Prof Francis Ofei | Dean, SMS, UCC and Chair of the Ghana STG |
| Dr. Marc Dzradosi | STG Review Team |
| Dr Lydia Dsane-Selby | NHIA |
| Dr Memuna Tanko | NHIA |
| Cecilia Senoo | Coalition of NGOs in Health |
| Dr Emmanuel Odame | MOH Policy Planning Monitoring and Evaluation (PPME) Directorate |
| Prof Alex Dodoo | College of Health Sciences, UG |
| Edith Annan | WHO Ghana Office |
| Ruby Awittor | Ghana Health Service. Ashiama District |
| Jennifer Manfo | GHS, Ashanti Regional Health Directorate |
| Dr Adwoa -Benneh | NHIA |
| Dr Berko Panyin Anto | FPPS, KNUST |
| George Hedidor | National Drug Resource Information Centre (NDIRC), MOH |

# References

1.	National Collaborating Centre for Chronic Conditions (UK). *Hypertension: Management in Adults in Primary Care: Pharmacological Update. (NICE Clinical Guidelines, No. 34.)*. 2006 03.07.2017]; Available from: <https://www.ncbi.nlm.nih.gov/books/NBK45886/>.

2.	Marfo, A.F. and F.T. Owusu-Daaku, *Evaluation of a pharmacist-led hypertension preventative and detection service in the Ghanaian community pharmacy: an exploratory study.* Int J Pharm Pract, 2016. **24**(5): p. 341-8.

3.	Ghana Statistical Service. *Population and Housing Census 2010*. 2012; Available from: <http://www.statsghana.gov.gh/docfiles/2010phc/Census2010_Summary_report_of_final_results.pdf>.

4.	Ghana Statistical Service (GSS), Ghana Health Service (GHS), and ICF International, *Ghana Demographic and Health Survey 2014 (GDHS)*. 2015: Rockville, Maryland, USA.

5.	World Health Organization (WHO). *Life tables by country- Ghana*. 2016 09.05.2017

]; Available from: <http://apps.who.int/gho/data/?theme=main&vid=60630>.

6.	Hippisley-Cox, J., et al., *Predicting cardiovascular risk in England and Wales: prospective derivation and validation of QRISK2.* British medical journal, 2008. **336**(7659): p. 1475-1482.

7.	Ettehad, D., et al., *Blood pressure lowering for prevention of cardiovascular disease and death: a systematic review and meta-analysis.* The Lancet, 2016. **387**(10022): p. 957-967.

8.	Singh, G.M., et al., *The age-specific quantitative effects of metabolic risk factors on cardiovascular diseases and diabetes: a pooled analysis.* PLoS One, 2013. **8**(7): p. e65174.
